# Examples of sub-millimeter, 7T, T_1_-weighted EPI datasets acquired with the T_1_23DEPI sequence

**DOI:** 10.1016/j.dib.2018.08.030

**Published:** 2018-08-16

**Authors:** Wietske van der Zwaag, Pieter F. Buur, Alessio Fracasso, Tessa van Doesum, Kâmil Uludağ, Maarten Versluis, José P. Marques

**Affiliations:** a Spinoza Centre for Neuroimaging, Amsterdam, Netherlands; b Institute of Neuroscience and Psychology, University of Glasgow, Glasgow G12 8QB, United Kingdom; c Maastricht University, Maastricht, Netherlands; d Philips Healthcare, Eindhoven, Netherlands; e Donders Institute for Brain, Cognition and Behaviour, Nijmegen, Netherlands

## Abstract

These imaging data are examples of sub-millimeter resolution T_1_-weighted EPI (Echo Planar Imaging) acquired using the T_1_23DEPI (T_1_-imaging with 2 3D-EPIs) sequence [1]; functional MRI data with matching resolution and distortion, and MP2RAGE (Magnetization Prepared 2 Rapid Acquisition Gradient Echoes) anatomical images [2], from the same subjects. Data from two protocols and subjects presented in the paper describing the sequence [1] are made available here:

1) 0.8 mm protocol: whole brain, axial T_1_23DEPI T_1_-weighted images; a 5-minute fMRI run with the same orientation and 27 mm coverage in the slice selection direction, covering the primary visual cortex. fMRI data were acquired while the volunteer viewed a flashing checkerboard stimulus; the unsmoothed GLM results of the fMRI and a 0.64 mm resolution MP2RAGE from the same subject. These data are from Experiment 3 in [1]

2) 0.7 mm protocol: partial brain T_1_23DEPI T_1_-weighted images with longer or shorter readouts; matching coronal echo planar images again acquired while viewing a flashing checkerboard stimulus and a 0.64 mm whole brain MP2RAGE from the same subject. These data are from Experiment 1 in  [1]

**Specifications Table**

**Table:** 

Subject area	Physics
More specific subject area	Medical imaging
Type of data	Magnetic Resonance Images (NIfTI format)
How data were acquired	Philips 7T MRI scanner
Data format	NIfTI files of: initial images, combined T_1_-weighted images, SPM functional results (Statistical Parametric Mapping, https://www.fil.ion.ucl.ac.uk/spm/)
Experimental factors	Initial MP2RAGE and T_1_23DEPI images were combined using the MP2RAGE image equation [2]. Functional data were motion corrected in SPM and a GLM (General Linear Model) analysis was run with the task described by a boxcar convolved with the canonical hrf. The 0.7 mm data were smoothed with a Gaussian of FWHM 0.7 mm prior to the GLM analysis to improve visualization of the results.
Experimental features	The T_1_23DEPI sequence is a version of the MP2RAGE where the GRE (Gradient Recalled Echo) readout has been replaced by a 3D-EPI readout. The possibility to acquire the 3D-EPI in blocks of several readouts (TFEPI or Turbo FLASH EPI in Philips language) allows optimizing the inversion times similarly as suggested for the MP2RAGE. Both the MP2RAGE and T_1_23DEPI data were acquired using the MISS (Multiple Instantaneously Switchable Scans) functionality provided by the scanner vendor.
Data source location	Data were acquired at the Spinoza Centre in Amsterdam
Data accessibility	Data are included in this article

**Value of the data**

• These data are an example of sub-millimeter fMRI data acquired with a 7 T MRI scanner. Researchers can use these to test their own co-registration or full laminar analysis pipelines. The whole brain MP2RAGE and T_1_23DEPI data have already been successfully run through the Freesurfer (https://surfer.nmr.mgh.harvard.edu/) pipeline, but many other pipelines remain to be tested.
• These data can also be used as an instruction/training dataset for researchers commencing work in the field of laminar imaging.
• The T_1_23DEPI data could be compared to VASO (Vascular Space Occupancy [3]) data acquired on the Siemens or Philips systems in terms of image quality; the imaging sequences are very similar although the optimal inversion times for blood nulling (in VASO) and T_1_-contrast (in T_1_23DEPI) differ.

## 1 Data

These fMRI data are examples of sub-millimeter fMRI data. The 0.7 and 0.8 mm data were both acquired on a 7T Philips system, along with echo planar images with T_1_ contrast (T_1_23DEPI) and MP2RAGE data as anatomical reference.

## 2 Experimental design, materials and methods

Both participants were healthy volunteers, scanned at 7T (Philips, Netherlands) with a 32-channel receive and 2-channel transmit volume coil (Nova Medical, USA) using the acquisition protocols specified below. Volunteers provided written informed consent prior to participating in the session. The protocol was approved by the ethics committee (METC) of the Amsterdam Medical Centre. All acquisition protocols for MP2RAGE and T_1_23DEPI were generated using the MISS (Multiple Instantaneously Switchable Scans) functionality on the Philips platform.

Both the MP2RAGE and T_1_23DEPI first and second inversion images were exported and subsequently combined in MATLAB (https://github.com/JosePMarques/MP2RAGE-related-scripts/). Because of the transmit-coil sensitivity map used in the SENSE (SENSitivity Encoding) reconstruction online, the combined phase images do not contain phase singularities and the combination can be done post-coil combination. In the MP2RAGE combination, an inversion efficiency of 0.96 was assumed.

The 0.8 mm data are from Experiment 3 in [1]. For the 0.8 mm datasets, the 3D-EPI parameters were: TE (echo time) = 25.5 ms, TR (repetition time) = 53 ms, EPI factor = 27, SENSE undersampling factor 4 (LR, Left-Right direction), slice oversampling factor 1.1, volume acquisition time 3.9 s. The FOV (Field of View) for this acquisition was not matched to that of the T_1_23DEPI, being only 27 mm in the HF direction (34 slices) while the T_1_23DEPI covered the entire brain. As the EPI factor, in-plane FOV, bandwidth and slice orientation remained the same, all distortions are equal between fMRI and T_1_23DEPI. For the functional run, the volunteer viewed a black and white checkerboard on a gray background alternating at 8 Hz, 10 s on, 20 s off, for 10 cycles [4]. The echo planar images (FUNC08.nii) and SPM t-score results (FUNC08SPM.nii) are provided.

Parameters for the T_1_23DEPI were: TR_T123DEPI_ 8.25 s, TI_1_/TI_2_ = 1000/3300 ms, α_1_/α_2_ = 19/15 degrees, TR = 53 ms, 20 readouts per block, total 12 blocks per volume. FOV = 170 * 189 * 120, voxel size 0.8 * 0.8 * 0.8 mm, 2 averages. The first and second inversion images are provided (T123DEPI08_INV1.nii; T123DEPI08_INV1ph; T123DEPI08_INV2.nii; T123DEPI08_INV2ph.nii) and the resulting T_1_-weighted image (T123DEPI08M.nii) and T_1_-map (T123DEPI08_T1M.nii). An intensity based mask of the second inversion was used to suppress the noise in the background of the T_1_-weighted image and the T_1_-map.

MP2RAGE data (MP2RAGE08.nii) were acquired with the following parameters: voxel size 0.64 mm isotropic, FOV 220 * 220 * 164 mm^3^, TR_MP2RAGE_/TE/TR_FLASH_ = 5.5 s/2.3 ms/6.2 ms, TI_1_/TI_2_ 800 ms/2700 ms, α_1_/ α_2_ = 7/5, t_acq_ 11 min.

The 0.7 mm data are from Experiment 1 in 0.7 [1]. In the 0.7 dataset two T_1_23DEPI׳s were acquired with different block lengths. T_1_23DEPI protocols with 3D-EPI readouts split into either 2 or 4 k-space sections (blocks) were acquired. A high-resolution (0.7 mm isotropic) 3D-EPI protocol previously used for sub-millimeter fMRI acquisitions [5] was used to acquire functional data and the T_1_23DEPI acquisition parameters were matched to its sequence parameters. The 3D-EPI protocol parameters were: FOV 120 * 131 * 24 mm^3^, matrix size 172 * 188 * 34, TR/TE = 57 ms/28 ms, EPI factor = 27, SENSE undersampling factor 3.5 (LR) * 1.3 (AP), slice oversampling factor 1.28, 68 EPI readouts (segments) per volume, volume acquisition time 3.9 s. No fat saturation was used. The stimulus was the same black and white checkerboard on a gray background, again alternating at 8 Hz, 10 s on, 20 s off, for 10 cycles [4]. The echo planar images (FUNC07.nii) and SPM t-score results (FUNC07SPM.nii) are provided.

The minimum inversion time of the first readout (TI_1_) in the 2-block protocol was limited by the length of the readout block. For the 2-block protocol this readout block length was 1.94 s, for the 4-block protocol it was 1.03 s. Parameters for the 2-block T_1_23DEPI were: TR_T123DEPI_ 10 s, TI_1_/TI_2_ = 1200/3800 ms, α_1_/α_2_ = 14/10 degrees, TR = 57 ms, 34 readouts per block, total 2 blocks per volume. FOV = 120 * 131 * 24, voxel size 0.7 * 0.7 * 0.7 mm, 4 averages, total acquisition time 80 s. The first and second inversion images are provided (T123DEPI07_bl2_INV1.nii; T123DEPI07_bl2_INV1ph.nii; T123DEPI07_bl2_INV2.nii; T123DEPI07_bl2_INV2ph.nii;) and the resulting T_1_-weighted image (T123DEPI07_bl2M.nii) and T_1_-map (T123DEPI07_bl2_T1M.nii). An intensity based mask of the second inversion image was used to suppress the noise in the background of the T_1_-weighted image and the T_1_-map.

Parameters for the 4-block T_1_23DEPI were: TR_T123DEPI_ 8.25 s, TI_1_/TI_2_ = 1000/2700 ms, α_1_/α_2_ = 20/16 degrees, TR = 57 ms, 18 readouts per block, total 4 blocks per volume. FOV = 120 * 131 * 24, voxel size 0.7 * 0.7 * 0.7 mm, 4 averages, total acquisition time 132 s. The first and second inversion images are provided (T123DEPI07_bl4_INV1.nii; T123DEPI07_bl4_INV1ph.nii; T123DEPI07_bl4_INV2.nii; T123DEPI07_bl4_INV2ph.nii;) and the resulting T_1_-weighted image (T123DEPI07_bl4M.nii) and T_1_-map (T123DEPI07_bl4_T1M.nii). An intensity based mask of the second inversion image was used to suppress the noise in the background of the T_1_-weighted image and the T_1_-map.

MP2RAGE data (MP2RAGE07.nii) were acquired with the following parameters: voxel size 0.64 mm isotropic, FOV 220 * 220 * 164 mm^3^, TR_MP2RAGE_/TE/TR_FLASH_ = 5.5 s/2.3 ms/6.2 ms, TI_1_/TI_2_ 800 ms/2700 ms, α_1_/ α_2_ = 7/5, t_acq_ 11 min. The readout block length of the MP2RAGE was 985ms, comparable to that of the 4-block protocol described above. For both the MP2RAGE and all T_1_23DEPI sequences a non-selective adiabatic inversion pulse (hyperbolic secant) was used.
